# COVID-19 vaccination intention and vaccine hesitancy among citizens of the Métis Nation of Ontario

**DOI:** 10.17269/s41997-023-00836-8

**Published:** 2024-01-08

**Authors:** Noel Tsui, Sarah A. Edwards, Abigail J. Simms, Keith D. King, Graham Mecredy, the Métis Nation of Ontario, Michael Schull, Joanne Meyer, Shelley Gonneville

**Affiliations:** 1 Métis Nation of Ontario, Ottawa, ON Canada; 2 Dalla Lana School of Public Health, University of Toronto, Toronto, ON Canada; 3 ICES Central, Toronto, ON Canada; 4 Faculty of Nursing, University of Alberta, Edmonton, AB Canada

**Keywords:** COVID-19 vaccines, Vaccine hesitancy, Métis, Indigenous health, Vaccins contre la COVID-19, réticence à l’égard de la vaccination, Métis, santé autochtone

## Abstract

**Objective:**

The study objective is to measure the influence of psychological antecedents of vaccination on COVID-19 vaccine intention among citizens of the Métis Nation of Ontario (MNO).

**Methods:**

A population-based online survey was implemented by the MNO when COVID-19 vaccines were approved in Canada. Questions included vaccine intention, the short version of the “5C” psychological antecedents of vaccination scale (confidence, complacency, constraint, calculation, collective responsibility), and socio-demographics. Census sampling via the MNO Registry was used achieving a 39% response rate. Descriptive statistics, bivariate analyses, and multinomial logistic regression models (adjusted for sociodemographic variables) were used to analyze the survey data.

**Results:**

The majority of MNO citizens (70.2%) planned to be vaccinated. As compared with vaccine-hesitant individuals, respondents with vaccine intention were more confident in the safety of COVID-19 vaccines, believed that COVID-19 is severe, were willing to protect others from getting COVID-19, and would research the vaccines (Confident OR = 19.4, 95% CI 15.5–24.2; Complacency OR = 6.21, 95% CI 5.38–7.18; Collective responsibility OR = 9.83, 95% CI 8.24–11.72; Calculation OR = 1.43, 95% CI 1.28–1.59). Finally, respondents with vaccine intention were less likely to let everyday stress prevent them from getting COVID-19 vaccines (OR = 0.47, 95% CI 0.42–0.53) compared to vaccine-hesitant individuals.

**Conclusion:**

This research contributes to the knowledge base for Métis health and supported the MNO’s information sharing and educational activities during the COVID-19 vaccines rollout. Future research will examine the relationship between the 5Cs and actual uptake of COVID-19 vaccines among MNO citizens.

## Résumé

**Objectif:**

Nous avons cherché à mesurer l’influence des antécédents psychologiques de vaccination sur l’intention de se faire vacciner contre la COVID-19 chez les citoyennes et citoyens de la Nation métisse de l’Ontario (NMO).

**Méthode:**

Un sondage populationnel en ligne a été mis en œuvre par la NMO quand des vaccins contre la COVID-19 ont été approuvés au Canada. Les questions posées ont porté sur l’intention de se faire vacciner, la version abrégée du modèle « 5C » de l’échelle de vaccination (Confiance, Contraintes, Complaisance, Calcul et responsabilité Collective) et le profil sociodémographique. Nous avons utilisé l’échantillonnage fondé sur le recensement via le registre de la NMO pour obtenir un taux de réponse de 39 %. Des statistiques descriptives, des analyses bivariées et des modèles de régression logistique multinomiale (ajustés selon les variables sociodémographiques) ont servi à analyser les données du sondage.

**Résultats:**

La majorité (70,2 %) des citoyennes et citoyens de la NMO prévoyaient se faire vacciner. Comparativement aux personnes réticentes à l’égard de la vaccination, les personnes ayant l’intention de se faire vacciner avaient plus confiance en l’innocuité des vaccins contre la COVID-19, considéraient la COVID-19 comme une maladie grave, étaient disposées à protéger les autres contre la COVID-19 et cherchaient à se renseigner au sujet des vaccins (Confiance : RC = 19,4, IC95% 15,5–24,2; Complaisance : RC = 6,21, IC95% 5,38–7,18; responsabilité Collective : RC = 9,83, IC95% 8,24–11,72; Calcul : RC = 1,43, IC95% 1,28–1,59). Enfin, les répondantes et les répondants ayant l’intention de se faire vacciner étaient moins susceptibles de laisser le stress quotidien les empêcher de se faire vacciner contre la COVID-19 (RC = 0,47, IC95% 0,42–0,53) comparativement aux personnes réticentes à l’égard de la vaccination.

**Conclusion:**

Cette étude contribue à la base de connaissances sur la santé des Métis et a appuyé les activités de sensibilisation et d’échange d’informations de la NMO pendant le déploiement des vaccins contre la COVID-19. Une étude future portera sur la relation entre les « 5C » et le recours réel aux vaccins contre la COVID-19 chez les citoyennes et citoyens de la NMO.

## Introduction

The COVID-19 pandemic is an unprecedented experience for individuals, families, and communities worldwide. In particular, racial/ethnic minority populations, low-income essential workers, and the Indigenous populations experienced a disproportionate impact from the COVID-19 pandemic due to pre-existing social determinants of health inequities (Huyser et al., 2022). COVID-19 vaccine uptake remains suboptimal at the population level and notably among individuals and communities at high risk for morbidity and mortality related to COVID-19, including Indigenous populations (Métis, Inuit, and First Nations) in Canada (Smylie et al., 2022; Statistics Canada, 2020). It remains crucial to address vaccine hesitancy, defined by the World Health Organization (WHO) as “the delay in the acceptance or refusal to vaccinate despite the availability of vaccine services” (Turner et al., 2021).

Indigenous Peoples in Canada are still experiencing the detrimental impacts of both historic and ongoing forms of colonization, including persistent health and economic inequalities, which were further exacerbated and widened during the COVID-19 pandemic (Sullivan et al., 2023). Heightened vaccine hesitancy among Indigenous Peoples stems from a long history of medical experimentation, involuntary sterilization, residential school experiences, and unethical research performed by institutions that promote vaccination (Sullivan et al., 2023). Therefore, recognizing these multifaceted factors when promoting vaccines to Indigenous Peoples is vital for uptake. Although research involving Indigenous Peoples is growing exponentially, research with Métis is still limited in scope, particularly in health research (Macdougall, 2017). Only two studies to date have examined the COVID-19 vaccines uptake in Métis, Inuit, and First Nations populations (Smylie et al., 2022; Statistics Canada, 2020). Statistics Canada, (2020) suggested a lower willingness among Métis to receive a COVID-19 vaccine compared to the non-Indigenous population in Canada. Only one qualitative study has examined vaccine hesitancy in Métis, and it was more than a decade ago during the H1N1 pandemic (Driedger et al., 2015). There remains a paucity of evidence on vaccine uptake, as well as exploring why uptake may be lower among Métis.

With a distinct heritage, culture, and language, the Métis are one of three Indigenous groups recognized in Canada (Métis Nation of Ontario | About the Métis of Ontario, n.d.). The Métis are the descendants of early unions between First Nations women and European men. The sole official registry of Métis people in Ontario is maintained by the Métis Nation of Ontario (MNO). Indigenous Peoples and their communities have historically benefited little from the majority of research involving Indigenous populations in Canada, which has been conducted by non-Indigenous researchers (Panel on Research Ethics, 2018). This study is led by the MNO, with two authors being Métis citizens and all authors having undergone Métis cultural training.

Given how little evidence existed on which to base Métis-specific health promotion efforts as the COVID-19 vaccines were rolling out, the MNO undertook a study that is the first of its kind to explore population-level vaccine uptake. This included the application of a validated scale developed and utilized to explore vaccine willingness and hesitancy, known as the “5C” scale (Betsch et al., 2018), to begin to understand vaccine acceptability and hesitancy in MNO citizens. The 5C scale provides an in-depth exploration of the psychological antecedents of vaccinations (Betsch et al., 2018). The scale assesses confidence (an individual’s trust in vaccines and the systems that provide them), complacency (the perceived risk of contracting and of the severity of the disease), constraint (structural and psychological barriers to vaccination), calculation (efforts in searching for information), and collective responsibility (willingness to protect others) (Betsch et al., 2018). The scale has been utilized to assess vaccine behaviour amid the currently evolving COVID-19 pandemic (Al-Sanafi & Sallam, 2021; Wismans et al., 2021). While it has been used with Indigenous populations in Canada, the approach taken has been broadly pan-Indigenous (Humble et al., 2021; Manca et al., 2022), with no specific application to the Métis population to date. This study aims to examine the impact of the 5C psychological antecedents on vaccination intention among MNO citizens.

## Methods

### Study design and setting

This study used an MNO-led, population-based online survey which collected information between February 8 and March 8, 2021. Since December 2020, Ontario has experienced a series of provincewide shutdowns and/or stay-at-home order (Ontario Newsroom, 2021). The province extended the stay-at-home order on February 8, 2021 (Ontario Newsroom, 2021). Subsequently, this mandate was lifted for Ontario regions outside of Toronto, Peel, and York on February 16 (Ranger, 2021). York region’s mandate was lifted on February 22, and mandates for Toronto and Peel regions were lifted on March 8, 2021 (Ranger, 2021). Please note that during the data collection period, Canada had just approved the use of several COVID-19 vaccines, with vaccination appointment bookings opening to adults over the age of 80 on March 1 (Ranger, 2021). Therefore, only a small percentage of the population would have received the COVID-19 vaccines during the data collection period.

At the time of the survey, Ontario had 23,377 registered MNO citizens (Métis Nation of Ontario, 2021a). To become an MNO citizen, applicants need to provide documents to support that they are ancestrally connected to the historic Métis Nation (Métis Nation of Ontario, 2021a).

Census sampling was used with all MNO citizens with a valid email on file. All eligible MNO citizens received an email invitation to the survey deployed through Qualtrics. All MNO citizens with a valid phone number on file also received an automated phone call about the online survey. The survey was also advertised through the MNO’s social media channels and website indicating MNO citizens would receive an invitation via email. All participants received a $5 coffee gift card upon completion of the survey and were entered into a draw for a chance to win one of 50 $100 VISA gift cards.

The survey included previously validated questions about vaccine perceptions (Betsch et al., 2018), sociodemographic variables, and general wellness. The survey was reviewed by three MNO senators, and MNO staff members and leadership. This study received ethical approval from the Sunnybrook Health Sciences Research Ethics Board (REB#3754).

### Measures

The primary outcome was MNO citizen’s vaccination intention. Respondents were asked the following: “Please let us know if you agree or disagree with the following statement: I plan to be vaccinated”, using a 5-point Likert scale (strongly agree to strongly disagree).

Independent variables of interest included the 5C psychological antecedents of vaccination: confidence, complacency, constraint, calculation, and collective responsibility. These antecedents were measured using the short form 5C scale (one to two items per antecedent) (Betsch et al., 2018). Survey questions used to measure the 5C psychological antecedents are presented in the Appendix. Vaccine hesitancy related to the 5Cs was assessed by dividing the respondents into three groups: intenders, i.e. respondents with a clear intent to receive the COVID-19 vaccines (strongly agree and agree responses); unsures, i.e. respondents who were hesitant (unsure response); and non-intenders, i.e. respondents who rejected the COVID-19 vaccines (strongly disagree and disagree responses). Confidence was measured through the respondents’ confidence toward the effectiveness and safety of the COVID-19 vaccines. Complacency was measured through the respondents’ perceived risk of contracting and of the severity of COVID-19. Constraint was measured through the respondents’ willingness to receive the COVID-19 vaccines despite barriers. Calculation was measured through the respondents’ engagement in researching and weighing the benefits and risks of receiving the COVID-19 vaccines. Collective responsibility was measured through the respondents’ belief as to whether everyone should be vaccinated to prevent the spread of COVID-19. These five antecedents provide insights into the individual’s mental representations and the attitudinal and behavioural tendencies that are influenced by their environment and the context in which they live (Betsch et al., 2018). The 5C questions also used a 5-point Likert scale, ranging from strongly agree (score = 5) to strongly disagree (score = 1) for responses. These were reduced to three categories for analyses: agree, neither agree nor disagree, and disagree.

Sociodemographic information, including gender (man; woman; non-binary or third gender or two-spirit), education (never been to school vs. attended primary or elementary school or attended secondary or high school; red seal or trade certificate; college or university degree, or graduate or professional degree), annual income (less than $24,999, $25,000–$49,999, $50,000–$74,999, $75,000–$99,999, $100,000 or more), and marital status (single; separated or divorced or widowed; in a relationship or married, living apart; or in a relationship or married, living together), was also collected. Age was linked from the MNO Registry to minimize the burden on the respondents and grouped into four categories (18–24, 25–44, 45–64, 65 +).

### Statistical analysis

Descriptive statistics were used to describe the sample of MNO citizens. The influence of non-response was explored for the sample using non-response survey weights developed based on age and sex of the overall MNO Registry. Weighted and unweighted estimates were very close (within 0.01–0.04%); therefore, all analyses are presented unweighted. Bivariate analyses were used to examine the association of the 5C psychological antecedents, as well as sociodemographic variables with COVID-19 vaccination intention. Furthermore, multinomial logistic regression models were used to describe the associations between the 5Cs and COVID-19 vaccination intention, adjusting for sociodemographic variables. Stratification by gender was performed where the groups were large enough. Data analysis was performed using SAS 9.4.

## Results

### Respondents

**Fig. 1:**
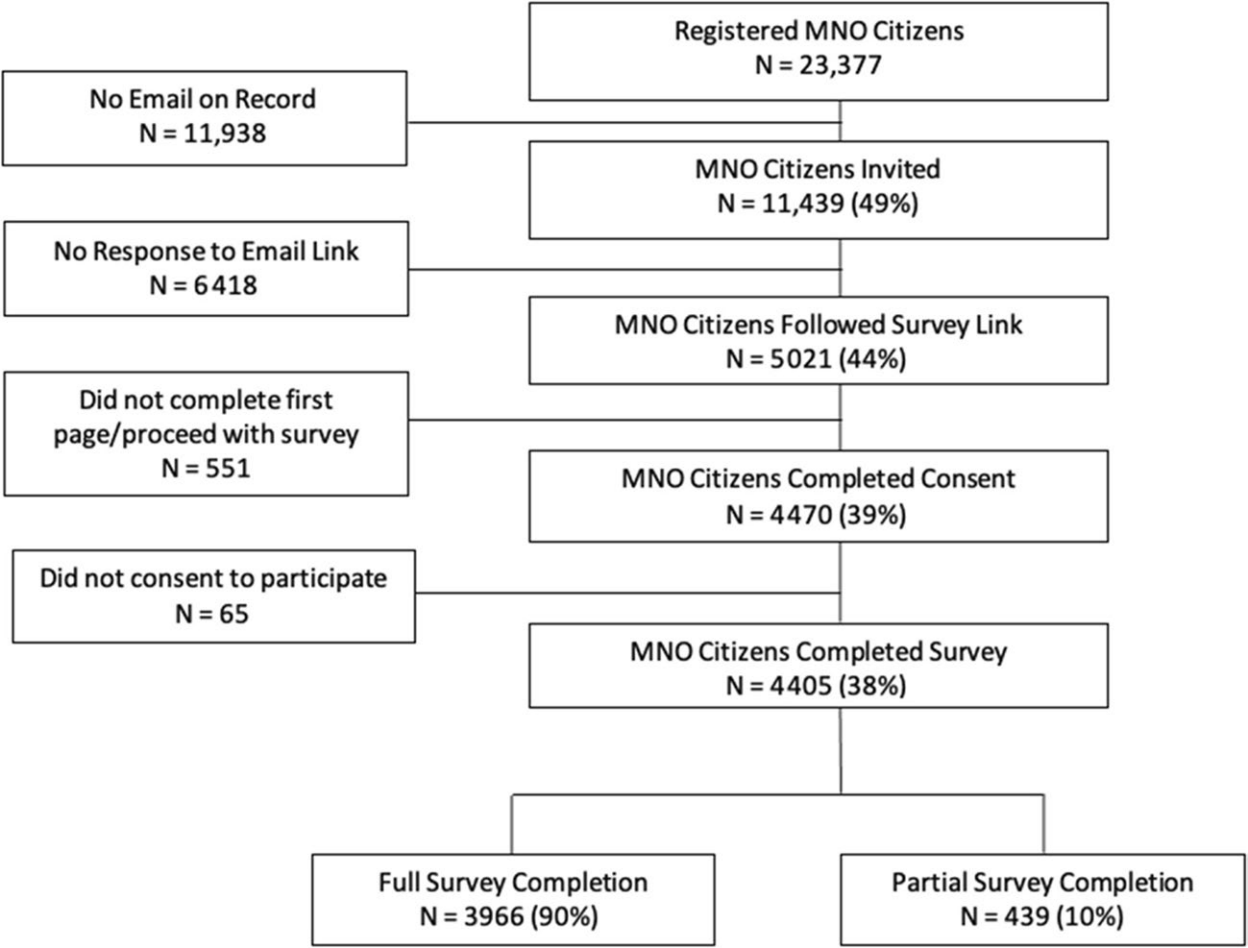
Breakdown of MNO citizens recruitment to complete the COVID-19 I* Survey deployed between February 8 and March 8, 2021. * Roman numeral referring to first of two surveys

There were 11,439 (49%) MNO citizens with an email on record during the study period. Of these, 5021 followed the survey link; 4470 completed consent and 38.5% (4405/11,439) completed the survey. Of the latter, 3966 respondents completed the full survey (Fig. 1).

### Respondent characteristics

The demographics of respondents are presented in Table 1. More than half of the respondents were women (54.6%). Most respondents were between 25 and 64 years old (74.9%). Most respondents were in a relationship or married (65.7%). Just under half (49.4%) of the respondents had at least some college education. Nearly 70% of respondents reported an annual household income of $50,000 or more.

**Table 1:** Demographic characteristics and COVID-19 vaccination intention rates of MNO citizens who responded to the “Please let us know if you agree or disagree with the following statement: I plan to be vaccinated” question (*N* = 4405) from the survey deployed between February 8 and March 8, 2021

COVID-19 vaccine intention	Plan to be vaccinated	%	Not sure	%	Do not plan to be vaccinated	%	*p*-value
Gender							0.0491
Woman	1597	69.5%	445	19.4%	256	11.1%	
Man	1328	72.4%	321	17.5%	185	10.1%	
Non-binary/third gender/two-spirit	21	65.5%	NR		NR		
Age							< 0.0001
18–24	254	72.4%	54	15.4%	43	12.3%	
25–44	884	62.4%	335	23.7%	197	13.9%	
45–64	1193	72.3%	293	17.8%	165	10.0%	
≥ 65	567	84.6%	73	10.9%	30	4.5%	
Highest level of education							0.0018
Never been to school; primary/elementary school or less; or secondary/high school	861	68.8%	245	19.6%	146	11.6%	
Red seal/trade certificate	203	64.0%	68	21.5%	46	14.5%	
College/university degree or graduate/professional degree	1888	73.0%	451	17.5%	246	9.5%	
Marital status							< 0.0001
Single	410	64.3%	141	22.1%	87	13.6%	
Separated or divorced/widowed	299	66.7%	100	22.3%	49	10.9%	
In a relationship/married, living apart	210	71.7%	60	20.5%	23	7.9%	
In a relationship/married, living together	2022	73.1%	471	17.0%	272	9.8%	
Annual household income							< 0.0001
Less than $9,999 per year or $10,000–$24,999 per year	354	63.1%	118	21.0%	89	15.9%	
$25,000–$49,999 per year	481	66.5%	151	20.9%	91	12.6%	
$50,000–$74,999 per year	520	70.0%	133	17.9%	90	12.1%	
$75,000–$99,999 per year	407	71.8%	110	19.4%	50	8.8%	
$100,000–$149,999 per year or over $150,000 per year	846	78.6%	151	14.0%	79	7.3%	

### COVID-19 vaccination intention

Overall, respondents indicated general positive attitudes toward the COVID-19 vaccines. A majority (70.2%) of respondents were intenders (51.0% strongly agreed and 19.2% agreed), 18.3% were unsures, and 10.5% were non-intenders (6.2% strongly disagreed and 4.3% disagreed). Most respondents aged 65 and above were intenders (84.6%), the highest among all age groups. Meanwhile, the age group between 25 and 44 had the lowest proportion of intenders (62.4%). A higher proportion of respondents living with a partner were intenders (73.1%) compared to single respondents (64.3%). Respondents with a college/university degree had the highest proportion of intenders (73.0%), and those with trade certificates had the lowest proportion of intenders (64.0%). For income levels, respondents reporting a household income higher than $100,000 had the highest proportion of intenders (78.6%) and those reporting an annual household income ranging from less than $9,999 to $24,999 had the lowest (63.1% of intenders).

### The 5C psychological antecedents

Intenders were significantly more likely to have confidence in COVID-19 vaccines compared to unsures or non-intenders (OR = 5.82, 95% CI 5.01–6.76 for unsures; and OR = 19.38, 95% CI 15.51–24.21 for non-intenders). Complacency was also higher in intenders, where they believe that COVID-19 is severe (OR = 2.64, 95% CI 2.36–2.95 for unsures; and OR = 6.21, 95% CI 5.38–7.18 for non-intenders). Intenders were more likely to weigh the risks and benefits to make the best-informed decision compared to non-intenders (OR = 1.43, 95% CI 1.28–1.59); however, there was no difference between intenders and unsures (OR = 1.02, 95% CI 0.92–1.13). Intenders were more likely to agree it is a collective action to prevent the spread of COVID-19 compared to unsures (OR = 4.41, 95% CI 3.84–5.06) or non-intenders (OR = 9.83, 95% CI 8.14–11.72). In contrast, constraint was lower in intenders, considering they do not believe that everyday stress will prevent them from receiving the COVID-19 vaccines (OR = 0.39, 95% CI 0.35–0.43 for unsures; and OR = 0.47, 95% CI 0.42–0.53 for non-intenders). When stratified by gender (man and woman), the associations were similar for both to the full cohort (data not presented) (Tables 2 and 3).

**Table 2:** Descriptive statistics of the 5C psychological antecedents and COVID-19 vaccination intention among MNO citizens

5C psychological antecedents	COVID-19 vaccination intention		
	Plan to vaccinate	Not sure	Do not plan to vaccinate
Confidence			
Strongly agree/agree	2332 (95.34%)	104 (4.25%)	10 (0.41%)
Neither agree nor disagree	513 (44.0%)	516 (44.25%)	137 (11.75%)
Strongly disagree/disagree	82 (16.14%)	143 (28.15%)	283 (55.71%)
Complacency			
Strongly agree/agree	2808 (80.05%)	547 (15.59%)	153 (4.36%)
Neither agree nor disagree	106 (26.43%)	171 (42.64%)	124 (30.92%)
Strongly disagree/disagree	50 (18.73%)	52 (19.48%)	165 (61.80%)
Constraints			
Strongly agree/agree	109 (50.93%)	66 (30.84%)	39 (18.22%)
Neither agree nor disagree	324 (41.27%)	320 (40.76%)	141 (17.96%)
Strongly disagree/disagree	2448 (80.37%)	363 (11.92%)	235 (7.72%)
Calculation			
Strongly agree/agree	2416 (71.82%)	649 (19.29%)	299 (8.89%)
Neither agree nor disagree	294 (61.76%)	104 (21.85%)	78 (16.39%)
Strongly disagree/disagree	200 (78.43%)	7 (2.75%)	48 (18.82%)
Collective responsibility			
Strongly agree/agree	2785 (81.74%)	492 (14.44%)	130 (3.82%)
Neither agree nor disagree	90 (18.67%)	231 (47.93%)	161 (33.40%)
Strongly disagree/disagree	46 (20.81%)	39 (17.65%)	136 (61.54%)

**Table 3:** Multiple linear regression models (unadjusted and adjusted for sociodemographic variables) for 5C psychological antecedents and COVID-19 vaccination intention among MNO citizens

5Cs/vaccination intention	Unadjusted					Adjusted				
	Plan to	Unsure (95% CI)		Do not plan to (95% CI)		Plan to	Unsure (95% CI)		Do not plan to (95% CI)	
		OR	*p*-value	OR	*p*-value		OR	*p*-value	OR	*p*-value
Confidence	Ref	6.03 (5.26–6.91)	< 0.0001	19.55 (16.00–23.91)	< 0.0001	Ref	5.82 (5.01–6.76)	< 0.0001	19.38 (15.51–24.21)	< 0.0001
Complacency	Ref	2.60 (2.35–2.87)	< 0.0001	5.50 (4.86–6.22)	< 0.0001	Ref	2.64 (2.36–2.95)	< 0.0001	6.21 (5.38–7.18)	< 0.0001
Constraints	Ref	0.38 (0.34–0.41)	< 0.0001	0.43 (0.38–0.48)	< 0.0001	Ref	0.39 (0.35–0.43)	< 0.0001	0.47 (0.42–0.53)	< 0.0001
Calculation	Ref	1.05 (0.97–1.15)	0.2444	1.47 (1.34–1.62)	< 0.0001	Ref	1.02 (0.92–1.13)	0.6829	1.43 (1.28–1.59)	< 0.0001
Collective responsibility	Ref	4.61 (4.07–5.22)	< 0.0001	10.01 (8.53–11.74)	< 0.0001	Ref	4.41 (3.84–5.06)	< 0.0001	9.83 (8.24–11.72)	< 0.0001

## Discussion

Using a population-based survey, our study demonstrated that the majority of MNO citizens planned to be vaccinated against COVID-19 as vaccine booking appointments were opening in early 2021. We also further sought to use validated measures to explore factors associated with COVID-19 vaccination intention, contributing one of the first population-based studies of vaccine hesitancy in Métis.

Overall, most respondents (70.2%) expressed high intentions to receive the COVID-19 vaccines as they were being released. The results from this study are in line with the existing Canadian report on vaccine hesitancy, where a majority of self-identified Métis (67.8%) reported a willingness to receive the COVID-19 vaccines (Statistics Canada, 2020). In a comparison with other Indigenous populations in Canada, our estimates were higher than those in studies that included a pan-Indigenous group, where 64.6% self-identified Indigenous respondents intended to receive the COVID-19 vaccines (Manca et al., 2022). In a comparison with the Canadian population, our estimates were on par with their high levels of vaccine intention, which ranged from 79.8% to 80% in surveys deployed in 2020 (Ogilvie et al., 2021; Tang et al., 2021). However, it is worth noting that Indigenous populations exhibited lower vaccine intention in studies conducted in 2020 (Ogilvie et al., 2021; Tang et al., 2021). With respect to other Indigenous populations worldwide, Native Americans/Alaskan natives had the highest vaccine refusal rate and the lowest level of institutional trust (Bagasra et al., 2021), and Native Hawaiian and other Pacific Islander populations demonstrated low vaccine uptake due to lack of trust in COVID-19 information (Juarez et al., 2022), indicative of low vaccine intention among these populations.

The actual vaccine uptake rates among MNO citizens have exceeded the already high rate of intended vaccination reported by MNO citizens participating in the survey. As of August 2021, 80% of MNO citizens had received at least one dose of COVID-19 vaccines, and 74% had received two doses (Métis Nation of Ontario, 2021b). Moreover, 87% of self-identified Métis reported receiving at least one dose of COVID-19 vaccines as of February 2022 in a Canada-wide survey (Government of Canada, 2022a). These rates are higher when compared to those from urban Indigenous populations (includes First Nations, Métis, and Inuit), where vaccine uptake rate of two doses of COVID-19 vaccines was 58.2% for urban Indigenous people living in Toronto as of December 2021 (Smylie et al., 2022). When compared with the vaccination trends within the broader Ontario population, the COVID-19 vaccine uptake rates among MNO citizens stand significantly higher. By August 2021, 70.3% of the Ontario population had received their first dose, and 62.2% had received two doses (Public Health Ontario, 2021). When compared to the Canadian population, the COVID-19 vaccine uptake rates among MNO citizens were also higher, where 71.4% of the Canadian population had received at least one dose of vaccines, and 61.9% had received two doses as of August 2021 (Government of Canada, 2022b). Overall, MNO citizens’ COVID-19 vaccine uptake rates have exceeded expectations and surpassed the vaccination rates of both the wider Ontario and Canadian populations. These accomplishments should be viewed through a strength-based lens, and the MNO community’s commitment to health and resilience should be emphasized.

Our study suggests MNO citizens who are intenders are confident in the effectiveness and safety of the COVID-19 vaccines. MNO citizens also consider COVID-19 to be severe. This is similar to the only other study to examine vaccine hesitancy in Métis people, where the perceived risk of contracting the disease was the biggest contributing factor to vaccination intention among Métis (Driedger et al., 2015). Moreover, our results align with those of Manca et al. (2022), where perceived risk of contracting COVID-19 and confidence in the safety of COVID-19 vaccines are contributing factors towards vaccine intention among Indigenous respondents. In addition, MNO citizens think they should protect others by receiving the COVID-19 vaccines and research the vaccines to weigh the benefits and risks in order to make the best-informed decision. Perceptions of the safety and effectiveness of the COVID-19 vaccines and a sense of collective responsibility have been shown to be associated with vaccination intention and uptake across a number of populations globally (Al-Sanafi & Sallam, 2021; Wismans et al., 2021). Misperceptions, like vaccines having high risks and low benefits, and mistrust in government or health authorities have also been identified as major drivers of vaccine hesitancy (Lazarus et al., 2022; Wismans et al., 2021). Although uptake was high in MNO citizens in the current context, the study sample was not the entire Métis Nation of Ontario population. These findings could inform ongoing efforts related to encouraging COVID-19 vaccine and booster uptake, as well as interventions for future vaccine rollouts and health promotion campaigns.

### Strengths and limitations of the study

A major strength of this study is that it was led by the MNO. The MNO was involved in the entire research process and dissemination materials were reviewed by the MNO prior to publication, which adhered to Indigenous data sovereignty principles (Kukutai & Taylor, 2016). Indigenous data sovereignty is the ability for Indigenous Peoples, communities, and Nations to be part of and have control over data collection, storage, ownership, access and consents, and application of data collected from and about them (Kukutai & Taylor, 2016). Indigenous data sovereignty and governance are essential rights protected by the United Nations Declaration on the Rights of Indigenous Peoples in Article 18–23 (Lovett et al., 2019), yet most public health research and government agencies continue to view the collection of data on Indigenous Peoples as servicing their own research or government requirements rather than supporting Indigenous Peoples’ development agendas (Kukutai & Taylor, 2016). Aside from supporting Indigenous data sovereignty, this study used the MNO Registry to conduct the largest population-based survey on vaccine hesitancy among Métis residing in Ontario. Another strength of this study is the application of the 5C scale, where the 5C scale offers a psychologically sound, validated, and reliable measure for vaccine behaviour (Betsch et al., 2018), and this study was the first to use the 5C scale to measure COVID-19 vaccine hesitancy among Métis populations.

There are a few limitations in this study. The first limitation is that this study only captured the COVID-19 vaccination intention of MNO citizens who have a valid email on file, which may have led to exclusion bias. When compared to the overall MNO citizen population, the survey respondents were representative in terms of age and sex with respect to the MNO Registry but could have differed by other important demographics not available for comparison, such as education level and annual income. In addition, the data collected are cross-sectional and only represent one point in time. This means the data represent only people registered with the MNO in January 2021, not all Métis people in Ontario. Thus, our findings may not be generalizable to those who are not registered MNO citizens. The cross-sectional nature of our data also represents a particular point in time of the COVID-19 vaccine rollout. Our data collection period fell under Ontario’s phase one of the vaccine rollout plan (Ranger, 2021), where only high-risk populations were eligible to receive the COVID-19 vaccines. As a result, this may have influenced respondents’ intention to receive the COVID-19 vaccines, considering the respondents’ kin network can have an impact on their vaccine behaviours (Driedger et al., 2015; Simms et al., 2023). In addition, the timing of the study could also have influenced the information shared by MNO citizens. Ontario was gradually lifting the stay-at-home mandate throughout the data collection period. Depending on the respondents’ residential location, the mandate may have been lifted as early as February 16 (8 days after survey deployment) or as late as March 8 (the last day of data collection). The vaccination intentions of the respondents and the 5Cs could have been very different if the survey was deployed during the later stage of vaccine rollout or not during a lockdown. The survey also did not collect information on Métis traditional knowledge or worldview on interacting with Western medicine, as well as family or personal experiences with racism within healthcare systems or government institutions. Both of these could potentially impact respondents’ willingness to engage with healthcare providers to obtain vaccination and to seek information about COVID-19 vaccines, as past experiences with healthcare services and family histories have been shown to influence vaccination decision (Mosby & Swidrovich, 2021; Turner et al., 2021). Last, we relied on self-reported willingness to receive a vaccine. Future research will examine the relationship between the 5Cs and the actual versus intended uptake of COVID-19 vaccines among MNO citizens.

## Conclusion

Among MNO citizens who planned to be vaccinated, there was a high level of confidence, complacency, and collective responsibility. Information on the relative importance of the psychological antecedents in MNO citizens assisted in real time to inform information sharing and educational activities tailored to MNO citizens during the COVID-19 vaccines rollout and enabled Métis-specific health promotion messaging. Given the paucity of evidence related to vaccine behaviour in Métis populations, this information can also be used to inform interventions for future vaccine rollouts and health promotion campaigns. Our study has also contributed to the knowledge base for Métis health, and future research will explore vaccination behaviour among MNO citizens in contexts outside of the COVID-19 pandemic.

## Contributions to knowledge

What does this study add to existing knowledge?

- There were no Métis-specific studies that looked at COVID-19 vaccine acceptability and hesitancy.
- This study was the first study to explore COVID-19 vaccine intention and hesitancy among the Métis Nation of Ontario (MNO) citizens.
- Ours was the first study to use the 5C psychological antecedents of vaccination to measure vaccine hesitancy in Métis populations.

What are the key implications for public health interventions, practice, or policy?

- The results were used for the MNO’s information sharing and educational activities for MNO citizens throughout the COVID-19 vaccine rollout.
- The results will also be used to inform interventions for future vaccine rollouts and to improve Métis health.
- Future research will examine the relationship between the 5Cs and the actual versus intended COVID-19 vaccine uptake among MNO citizens.

## Data Availability

Given the tension that releasing open data would create for the Métis Nation of Ontario (MNO) as all governing members from the Métis Nation walk the path towards self-government and self-determination, the data will not be available to everyone and will remain in the control of the MNO. Researchers interested in supporting Métis health research in collaboration with the MNO may request access and get approval for use.

## Appendix

Statements used to measure 5C psychological antecedents

## References

[CR1] Al-Sanafi M, Sallam M (2021). Psychological determinants of COVID-19 vaccine acceptance among healthcare workers in Kuwait: A cross-sectional study using the 5C and vaccine conspiracy beliefs scales. Vaccines.

[CR2] Bagasra AB, Doan S, Allen CT (2021). Racial differences in institutional trust and COVID-19 vaccine hesitancy and refusal. BMC Public Health.

[CR3] Betsch, C., Schmid, P., Heinemeier, D., Korn, L., Holtmann, C., & Böhm, R. (2018). Beyond confidence: Development of a measure assessing the 5C psychological antecedents of vaccination. *In**PLoS ONE, 13*(12). 10.1371/journal.pone.0208601 10.1371/journal.pone.0208601 PMC6285469 30532274

[CR4] Driedger, S. M., Maier, R., Furgal, C., & Jardine, C. (2015). Factors influencing H1N1 vaccine behavior among Manitoba Metis in Canada: A qualitative study. *BMC Public Health*, *15*(1). 10.1186/s12889-015-1482-2 10.1186/s12889-015-1482-2 PMC4334920 25884562

[CR5] Government of Canada. (2022a). *COVID-19 vaccination coverage by ethnicity: Insight from the Canadian Community Health Survey (CCHS)*. https://www.canada.ca/en/public-health/services/immunization-vaccines/vaccination-coverage/covid-19-vaccination-coverage-ethnicity-insight-canadian-community-health-survey.html. Accessed 8 Aug 2023

[CR6] Government of Canada. (2022b). *COVID-19 vaccination coverage in Canada - Canada.ca*. Government of Canada. https://health-infobase.canada.ca/covid-19/vaccination-coverage/archive/2021-08-13/. Accessed 8 Aug 2023

[CR7] Humble RM, Sell H, Dubé E, MacDonald NE, Robinson J, Driedger SM, Sadarangani M, Meyer SB, Wilson S, Benzies KM, Lemaire-Paquette S, MacDonald SE (2021). Canadian parents’ perceptions of COVID-19 vaccination and intention to vaccinate their children: Results from a cross-sectional national survey. Vaccine.

[CR8] Huyser, K. R., Yellow Horse, A. J., Collins, K. A., Fischer, J., Jessome, M. G., Ronayne, E. T., Lin, J. C., Derkson, J., & Johnson-Jennings, M. (2022). Understanding the associations among social vulnerabilities, indigenous peoples, and COVID-19 cases within Canadian Health Regions. *International Journal of Environmental Research and Public Health*, *19*(19). 10.3390/ijerph191912409 10.3390/ijerph191912409 PMC9566440 36231708

[CR9] Juarez R, Phankitnirundorn K, Ramirez A, Peres R, Maunakea AK, Okihiro M (2022). Vaccine-associated shifts in SARS-CoV-2 infectivity among the Native Hawaiian and other Pacific Islander population in Hawaii. American Journal of Public Health.

[CR10] Kukutai, T., & Taylor, J. (2016). Data sovereignty for Indigenous peoples: Current practice and future needs. In *Indigenous Data Sovereignty: Toward an Agenda* (pp. 1–23). ANU Press.

[CR11] Lazarus JV, Wyka K, White TM, Picchio CA, Rabin K, Ratzan SC, Parsons Leigh J, Hu J, El-Mohandes A (2022). Revisiting COVID-19 vaccine hesitancy around the world using data from 23 countries in 2021. Nature Communications.

[CR12] Lovett, R., Lee, V., Kukutai, T., Cormack, D., Rainie, S., & Walker, J. (2019). Good data practices for Indigenous data sovereignty and governance. In *Institute of Network Cultures*. 10.3233/SJI-161023

[CR13] Macdougall, B. (2017). *Land, family and identity: contextualizing Metis health and well-being* (pp. 5–23). Prince George: National Collaborating Centre for Aboriginal Health.

[CR14] Manca, T., Humble, R. M., Aylsworth, L., Cha, E., Wilson, S. E., Meyer, S. B., Greyson, D., Sadarangani, M., Parsons Leigh, J., & MacDonald, S. E. (2022). “We need to protect each other”: COVID-19 vaccination intentions and concerns among racialized minority and Indigenous Peoples in Canada. *Social Science and Medicine*, *313*(November), 115400. https://www.sciencedirect.com/science/article/pii/S0277953622007067?via%3Dihub. Accessed 1 Sep 2023 10.1016/j.socscimed.2022.115400 PMC9519366 36206660

[CR15] *Métis Nation of Ontario | About the Métis of Ontario*. (n.d.). Retrieved September 17, 2021, from https://www.metisnation.org/about-the-mno/. Accessed 17 Sep 2021

[CR16] Métis Nation of Ontario. (2021a). *Métis Nation of Ontario Registry and Self-Government Readiness Review Final Report*. https://www.metisnation.org/wp-content/uploads/2021/06/FINAL-Know-History-Report-re-MNO-Registry-and-Self-Government-Readiness-Review-w-appendices.pdf

[CR17] Métis Nation of Ontario. (2021b). *New milestone in Métis vaccination: 80% of citizens have received first dose - Métis Nation of Ontario*. https://www.metisnation.org/news/new-milestone-in-metis-vaccination-80-of-citizens-have-received-first-dose/

[CR18] Mosby I, Swidrovich J (2021). Medical experimentation and the roots of COVID-19 vaccine hesitancy among Indigenous Peoples in Canada. CMAJ.

[CR19] Ogilvie GS, Gordon S, Smith LW, Albert A, Racey CS, Booth A, Gottschlich A, Goldfarb D, Murray MCM, Galea LAM, Kaida A, Brotto LA, Sadarangani M (2021). Intention to receive a COVID-19 vaccine: Results from a population-based survey in Canada. BMC Public Health.

[CR20] Ontario Newsroom. (2021). *Ontario extending stay-at-home order across most of the province to save lives*. Ontario Newsroom. https://news.ontario.ca/en/release/60261/ontario-extending-stay-at-home-order-across-most-of-the-province-to-save-lives. Accessed 15 Aug 2023

[CR21] Panel on Research Ethics. (2018). *Tri-Council Policy Statement: Ethical Conduct for Research Involving Humans – TCPS 2 (2018) – Chapter 9: Research Involving the First Nations, Inuit and Métis Peoples of Canada*. TCPS2. https://ethics.gc.ca/eng/tcps2-eptc2_2018_chapter9-chapitre9.html. Accessed 30 Aug 2023

[CR22] Public Health Ontario. (2021). Ontario COVID-19 data tool | Public Health Ontario. In *Public Health Ontario*. https://www.publichealthontario.ca/en/Data-and-Analysis/Infectious-Disease/COVID-19-Data-Surveillance/COVID-19-Data-Tool?tab=vaccine. Accessed 16 Aug 2023

[CR23] Ranger, M. (2021). *Timeline: A year of pandemic life in Toronto*. CityNews Toronto. https://toronto.citynews.ca/2021/03/11/timeline-a-year-of-pandemic-life/. Accessed 15 Aug 2023

[CR24] Simms, A. J., King, K. D., Tsui, N., Edwards, S. A., & Mecredy, G. (2023). COVID-19 vaccine behaviour among citizens of the Métis Nation of Ontario: A qualitative study. *Vaccine*, *41*(38), 5640–5647. https://www.sciencedirect.com/science/article/pii/S0264410X23008915?via%3Dihub. Accessed 2 Sep 2022 10.1016/j.vaccine.2023.07.060 37550144

[CR25] Smylie J, McConkey S, Rachlis B, Avery L, Mecredy G, Brar R, Bourgeois C, Dokis B, Vandevenne S, Rotondi MA (2022). Uncovering SARS-COV-2 vaccine uptake and COVID-19 impacts among First Nations, Inuit and Métis Peoples living in Toronto and London, Ontario. CMAJ: Canadian Medical Association Journal = Journal de l’Association Medicale Canadienne.

[CR26] Statistics Canada. (2020). *StatCan COVID-19* *: Data to insights for a better Canada*. *45280001*, 1–9. https://www150.statcan.gc.ca/n1/pub/45-28-0001/2021001/article/00011-eng.htm. Accessed 17 Sep 2022

[CR27] Sullivan, P., Starr, V., Dubois, E., Starr, A., Acharibasam, J. B., & McIlduff, C. (2023). Where past meets present: Indigenous vaccine hesitancy in Saskatchewan. *Medical Humanities*, medhum-2022–012501. 10.1136/medhum-2022-012501 10.1136/medhum-2022-012501 PMC10439261 36604166

[CR28] Tang X, Gelband H, Nagelkerke N, Bogoch II, Brown P, Morawski E, Lam T, Jha P (2021). COVID-19 vaccination intention during early vaccine rollout in Canada: A nationwide online survey. The Lancet Regional Health - Americas.

[CR29] Turner PJ, Larson H, Dubé È, Fisher A (2021). Vaccine hesitancy: Drivers and how the allergy community can help. Journal of Allergy and Clinical Immunology: In Practice.

[CR30] Wismans, A., Thurik, R., Baptista, R., Dejardin, M., Janssen, F., & Franken, I. (2021). Psychological characteristics and the mediating role of the 5C model in explaining students’ COVID-19 vaccination intention.* PLoS ONE, 16*(8), e0255382. 10.1371/journal.pone.0255382 10.1371/journal.pone.0255382 PMC8357093 34379648

